# Outpatient care for facial palsy—a survey on patient satisfaction in uni- and interdisciplinary approaches

**DOI:** 10.3389/fneur.2024.1354583

**Published:** 2024-02-07

**Authors:** Kathrin Machetanz, Linda Oberle, Sophie S. Wang, Eliane Weinbrenner, Mykola Gorbachuk, Henrik Lauer, Adrien Daigeler, Marcos Tatagiba, Georgios Naros, Ruth C. Schäfer

**Affiliations:** ^1^Department of Neurosurgery and Neurotechnology, Eberhard Karls University, Tuebingen, Germany; ^2^Department of Hand, Plastic, Reconstructive and Burn Surgery, BG Unfallklinik Tuebingen, Tuebingen, Germany

**Keywords:** Bell’s Palsy, facial palsy, patient satisfaction, quality of life, vestibular schwannoma

## Abstract

**Objective:**

The various causes of facial palsy, diagnostic methods and treatment approaches frequently involve different medical specialities. Nevertheless, there exist only few specialized consultation and therapy services for patients with facial palsy (FP) in Germany. The aim of the present study was to evaluate factors affecting quality of life (QoL) and treatment satisfaction of patients presenting to an interdisciplinary facial nerve outpatient clinic.

**Methods:**

The study analyzed patients presenting to the interdisciplinary facial palsy outpatient clinic in Tuebingen between February 2019 and December 2022. General satisfaction and QoL was estimated by numerous self-rating questionnaires: ZUF-8, SF-36, FDI, FaCE, PHQ-9. An ANOVA was performed to analyze determinants affecting the ZUF-8. Correlation analyses between cause and regeneration of FP as well as questionnaire scores were performed. Results were compared with a group of patients who were managed in an unidisciplinary setting.

**Results:**

In total, 66 patients with FP were enrolled. FP patients showed increased levels of depression (PHQ-9: 14.52 ± 3.8) correlating with recovery of the palsy (*p* = 0.008), FaCE (*p* < 0.001) and FDI ratings (*p* < 0.001). There was a high level of satisfaction with the services provided during the uni-and interdisciplinary consultation (ZUF-8: 24.59 ± 6.2), especially among the 12/66 patients who received reconstructive, surgical treatment. However, some patients requested more psychological and ophthalmological support.

**Conclusion:**

High levels of treatment satisfaction can be achieved in both an uni-and interdisciplinary setting. However, multimodal therapy approaches should be applied, considering physical and psychological aspects. In the absence of recovery, surgical interventions must be considered as treatment options. Further studies should continue to investigate potential differences between uni-and interdisciplinary treatment.

## Introduction

Temporary and permanent facial palsy (FP) might be induced by a broad spectrum of disorders, including stroke, tumor, trauma, iatrogenic (e.g., resection of vestibular schwannomas or tumors of the parotid gland) or idiopathic paralysis (i.e., Bell’s Palsy) (1, 2). It is a debilitating and highly visible condition that can lead to physical complaints (e.g., corneal irritation, problems with drinking) and psychological problems due to appearance-affecting facial asymmetry and resulting stigmatization (3–7). In skull base surgery, the current EANO guidelines for vestibular schwannomas therefore more often recommend irradiation or—in case of large tumors—partial resection followed by irradiation to prevent FP (8, 9). However, previous studies also demonstrated that residual tumor can affect patient’s quality of life (QoL) or increase the risk of recurrence (10–12). Recurrence surgery, in turn, increases the risk of FP in comparison to primary resection. Furthermore, potential recovery of the facial nerve should not be underestimated. Improvement of facial function may occur up to approximately 1.5 years after the onset of FP, and depends on, e.g., the cause and severity of the palsy as well as effectiveness of medical support (13–18). Consequently, in addition to preventing the occurrence of FP, there should be an increased effort to encourage specialized treatment options for facial rehabilitation.

FP patients are likely to have contact with numerous medical specialties and disciplines. Neurologists are often the initial patient contact in new, non-iatrogenic FP. Conservative therapy is often guided by speech therapists and physiotherapists. Neurosurgeons and otolaryngologists are involved in the treatment of iatrogenic FP after, e.g., the resection of parotid tumors or vestibular schwannomas. In case conservative treatment is not satisfactory, ophthalmologists, maxillofacial and plastic surgeons are involved, e.g., in the treatment of lagophthalmos. However, therapists and physicians are often not specialized in FP, as it is only a minor topic of their specialty. Moreover, a standardized therapy does not exist: There are numerous physiotherapeutic approaches (e.g., Bobath, Proprioceptive Neuromuscular Facilitation) (19), that often originate from extremity rehabilitation and are adapted to the face. For surgical procedures, ENT surgeons mainly use direct (facio-facial and hypoglossal-facial) extracranial reconstruction techniques, whereas neurosurgeons are more familiar with intracranial facio-facial and/or hypoglossal-facial nerve reconstruction (20). In contrast, maxillofacial and plastic surgeons mostly use masseteric-facial and cross-face reconstructions in FP (21). This unidisciplinary segregation can imply that patients (i) receive a therapy that is not the most suitable for them, but the one that their physician is familiar with, or (ii) that they have to pass through a series of medical appointments and travel long distances to get there. A previous study analyzing the health service situation for FP patients in Great Britain demonstrated that in total 10% of the patients had to travel >115 miles to receive specific FP therapy (22). In Germany, there are also only a few interdisciplinary healthcare services that are specifically established for patients with FP.

In this context, the present survey study evaluated uni-and interdisciplinary outpatient services for patients with facial palsy. A set of self-rating questionnaires—which in the past have shown high reliability in predicting generic QoL (i.e., SF-36), depression (i.e., PHQ-9), facial function-associated QoL (i.e., FaCE and FDI), and treatment satisfaction (i.e., ZUF-8) (23–27), were used to investigate factors impacting QoL and treatment satisfaction in patients with FP. The objective of these analyses is to improve future patient management of patients with FP and, thus, contribute to an improvement in facial recovery and quality of life for patients.

## Methods

### Patients

In this prospective study 108 patients with FP were contacted and invited to participate by answering standardized questionnaires on QoL, demographics and treatment satisfaction. All adult and native German speaking patients who were treated in the interdisciplinary facial outpatient clinic (INTER; conducted by the Department of Neurosurgery and Plastic Surgery Tuebingen) and a control group of patients who were treated due to FP at the University Hospital of Tuebingen in an unidisciplinary setting (UNI; e.g. at the Department of Neurology or Neurosurgery) were contacted. The latter were identified using a computer-based search of the hospital system (i.e., SAP) based on the ICD-10 code G.51.0 and assessment of the medical reports. Inclusion criteria were (i) age over 18 years, and (ii) facial palsy as reason for presentation to the hospital. Exclusion criteria were (i) the presence of central FP (i.e., stroke, multiple sclerosis), (ii) recurrent occurrence of FP, and (iii) documentation of manifest cognitive impairment. The study was carried out in accordance with the recommendations of the ethics committee of the Eberhard Karls University Tuebingen.

### Questionnaires

Several questionnaires were completed by the participants to examine demographics, treatment satisfaction and QoL of patients with FP: a self-designed questionnaire on patient characteristics and general aspects of therapy, the adapted Client Satisfaction Questionnaire (ZUF-8) to evaluate treatment satisfaction, the general Short-Form Health Survey (SF-36) and Patient Health Questionnaire-9 (PHQ-9) to analyze general QoL and depression, and finally the Facial Disability Index (FDI) and Facial Clinimetric Evaluation (FaCE) for evaluation of facial function specific QoL.

The ZUF-8 is a German adaptation of the Client Satisfaction Questionnaire (CSQ) and evaluates subjective patient satisfaction through 8 items (28). It is often used for evaluation and quality assurance of treatments. The response options are classified on a Likert scale from 1 to 4. During the collection 4/8 items are negatively poled and have to be reversed during the evaluation, so that patients can award overall a minimum of 8 (low satisfaction) and a maximum of 32 (high satisfaction) points. In somatic health services, a cut-off value of 24 points was identified for a *“satisfactory treatment”* (29).

The SF-36 is a very common and extensively researched instrument designed to measure health-related QoL (30, 31). Grouped into the two main categories “mental health” and “physical health,” its 36 items can be divided into eight distinct health domains: vitality/energy (SF36-VT), social functioning (SF36-SF), role limitations due to emotional problems (SF36-RE) and mental health/psychological distress (SF36-MH), physical functioning (SF36-PF), role limitation due to physical health problems (SF36-RP), bodily pain (SF36-BP), general health perceptions (SF36-GH) (6). Each of the eight domain is scored from 0 to 100, with a lower score corresponding to a lower QoL.

The PHQ-9 consists of nine items and is a well-validated instrument for identifying and assessing the severity of depression in individuals (24). PHQ-9 items are rated based on frequency of occurrence in the past 2 weeks with 0: not at all, 1: several days, 2: more than half of the days or 3: nearly every day. Item scores are summed to a total score ranging from 0 to 27 with higher scores indicating a more severe depression.

The FDI is a patient-reported assessment tool to evaluate the impact of FP on QoL including physical limitations and emotional wellbeing (25). It contains of 10 items with response options from 1 to 5, which can be grouped into the two main categories physical function (−25 = worst to 100 = best function; FDI-PF) and social function (0 = worst to 100 = best function; FDI-SF).

The FaCE is an outcome measure consisting of 15 questions which are answered by the patient on a 5-point Likert scale (1-lowest level of function, 5-highest level of function) (32, 33). It evaluates the facial function in 6 domains: facial movement, facial comfort, eye comfort, oral function, lacrimal control and social function. Domain scores are transformed to a total score from 100 (best) to 0 (worst).

### Statistics

Statistical tests were performed using SPSS (IBM SPSS Statistics for Windows, Version 26.0. Armonk, NY: IBM Corp.). Group differences of clinical characteristics (e.g., sex, patient’s age, distance to home) were evaluated by Chi-squared or Kruskal-Wallis tests. An univariate ANOVA was performed to analyze potential determinants affecting the ZUF-8. Correlation analyses were performed by Spearman’s correlation. Data are shown as mean ± SD. Statistical significance was considered at *p* < 0.05 for each statistical test.

## Results

### Patient characteristics—INTER vs. UNI

**Figure 1:**
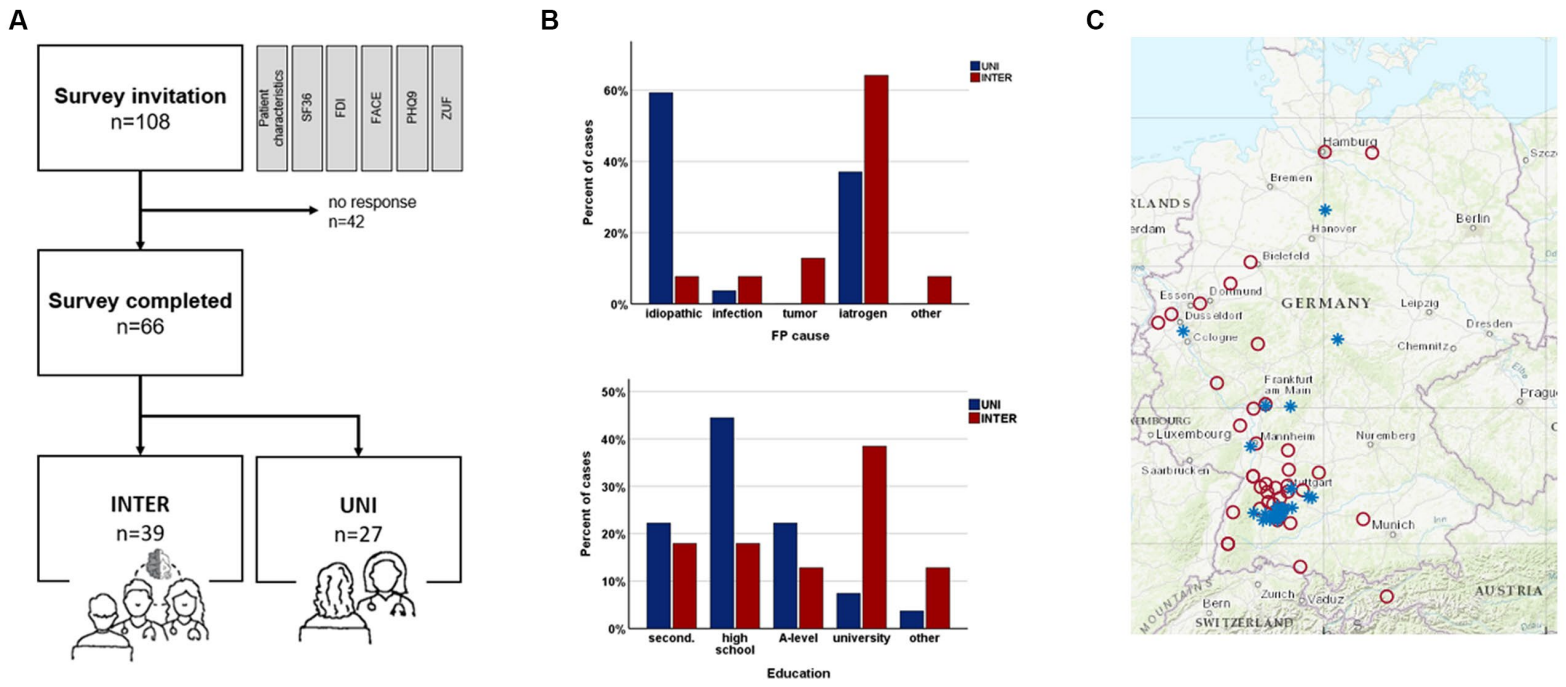
Overview about differences between INTER und UNI. **(A)** Flow chart about the surveys and the number of patients included into the groups. **(B)** Group differences of education level and cause of facial palsy. **(C)** Distribution of patients’ places of residence; blue, UNI; red, INTER.

A total of 108 questionnaires were submitted. Overall, 66/108 (61.1%) patients returned completed surveys, resulting in 39/66 (59.1%) questionnaires from the INTER group (response rate: 39/56, 69.6%) and 27/66 (40.9%) from the UNI group (response rate: 27/52, 51.9%) (Table 1). The mean age of the total cohort was 54.2 ± 13.5 years and did not differ between the two groups. However, there were differences in sex distribution and education level between the groups, with more women and significantly more university degrees in the INTER group than in the UNI group. The majority (21/39, 53.8%) of the INTER group had already been treated in Tuebingen and had thus become aware of the facial outpatient service, while 3/39 (7.7%) had been informed about the consultation by their neurologist or general practitioner and 5/39 (12.8%) by the Internet. However, 10/39 (25.6%) patients did not provide information about their source of information. The catchment area for the INTER consultation was significantly larger than for the UNI group (Figure 1).

**Table 1:** Patient characteristics.

	Total	INTER	UNI		Idiopathic	Iatrogen	
	*n* = 66	*n* = 39	*n* = 27		*N* = 19	*N* = 35	
Sex							
Male	23/66 (34.8%)	8 (20.5%)	15 (55.6%)	*X*^2^ = 8.63	10 (52.6%)	9 (25.7%)	*X*^2^ = 3.91
Female	43/66 (65.2%)	31 (79.5%)	12 (44.4%)	*p* = 0.003*	9 (47.4%)	26 (74.3%)	*p* = 0.048*
Age	54.2 ± 13.5	52.7 ± 12.4	56.3 ± 14.6	*p* = 0.418	58.63 ± 14.5	54.66 ± 11.5	*p* = 0.441
Marital status							
Single	8 (12.1%)	4 (10.3%)	4 (14.8%)	*X*^2^ = 1.90	1 (5.3%)	4 (11.4%)	*X*^2^ = 2.55
Married/partnership	50 (75.8%)	31 (79.5%)	19 (70.4%)	*p* = 0.592	14 (73.7%)	27 (77.1%)	*p* = 0.467
Divorced	7 (10.6%)	4 (10.3%)	3 (11.1%)		3 (15.8%)	4 (11.4%)	
Widowed	1 (1.5%)	0 (0%)	1 (3.7%)		1 (5.3%)	0 (0%)	
Education							
Secondary school^#^	13 (19.7%)	7 (17.9%)	6 (22.2%)	*X*^2^ = 12.32	3 (15.8%)	7 (20.0%)	*X*^2^ = 8.70
High school^##^	19 (28.8%)	7 (17.9%)	12 (44.4%)	*p* = 0.015*	10 (52.6%)	7 (20.0%)	*p* = 0.069
A-level	11 (16.7%)	5 (12.8%)	6 (22.2%)		4 (21.1%)	6 (17.1%)	
University	17 (25.8%)	15 (38.5%)	2 (7.4%)		2 (10.5%)	12 (34.3%)	
Other	6 (9.0%)	5 (12.9%)	1 (3.7%)		0 (0%)	3 (8.6%)	
Distance to home (km)	151.9 ± 175.8	189.5 ± 182.4	97.5 ± 153.1	*H* = 9.96	39.6 ± 54.0	207.8 ± 185.0	*H* = 17.00
				*p* = 0.002*			*p* < 0.001*
VS surgery							
No	26 (39.4%)	11 (28.2%)	15 (55.6%)	*X*^2^ = 4.99			
Yes	40 (60.6%)	28 (71.8%)	12 (44.4%)	*p* = 0.025*			
Outpatient clinic							
INTER					3 (15.8%)	25 (71.4%)	*X*^2^ = 15.27
UNI					16 (84.2%)	10 (28.6%)	*p* < 0.001*
FP side							
Left	29 (43.9%)	20 (51.3%)	9 (33.3%)	*X*^2^ = 3.21	5 (26.3%)	18 (51.4%)	*X*^2^ = 3.18
Right	36 (54.5%)	19 (48.7%)	17 (63%)	*p* = 0.201	14 (73.7%)	17 (48.6%)	*p* = 0.204
Bilateral	1 (1.5%)	0 (0%)	1 (3.7%)		0 (0%)	0 (0%)	
FP cause							
Idiopathic	19 (28.8%)	3 (7.7%)	16 (59.3%)	*X*^2^ = 22.90			
Infection	4 (6.1%)	3 (7.7%)	1 (3.7%)	*p* < 0.001*			
Tumor	5 (7.6%)	5 (12.8%)	0 (0%)				
Iatrogen	35 (53.0%)	25 (64.1%)	10 (37.0%)				
Other	3 (4.5%)	3 (7.7%)	0 (0%)				
FP duration							
≤1 year	20 (30.3%)	3 (7.7%)	17 (63.0%)	*X*^2^ = 34.05	9 (47.4%)	9 (25.7%)	*X*^2^ = 8.41
1–2 years	12 (18.2%)	5 (12.8%)	7 (25.9%)	*p* < 0.001*	5 (26.3%)	4 (11.4%)	*p* = 0.135
2–3 years	10 (15.2%)	10 (25.6%)	0 (0%)		1 (5.3%)	7 (20.0%)	
3–4 years	5 (7.6%)	5 (12.8%)	0 (0%)		0 (0%)	4 (11.4%)	
4–5 years	6 (9.1%)	4 (10.3%)	2 (7.4%)		2 (10.5%)	3 (8.6%)	
>5 years	13 (19.7%)	12 (30.8%)	1 (3.7%)		2 (10.5%)	8 (22.9%)	
FP improvement							
100%		0 (0%)	10 (37.0%)	*X*^2^ = 24.77	8 (42.1%)	1 (2.9%)	*X*^2^ = 16.38
>75%		8 (20.5%)	10 (37.0%)	*p* < 0.001*	6 (31.6%)	10 (28.6%)	*p* = 0.006*
50-75%		10 (25.6%)	1 (3.7%)		1 (5.3%)	7 (20.0%)	
<50%		13 (33.3%)	4 (14.8%)		2 (10.5%)	13 (37.1%)	
No improvement		7 (17.9%)	2 (7.4%)		2 (10.5%)	4 (11.4%)	
Missing data		1 (2.6%)	0 (0%)		0 (0%)	0 (0%)	
FDI							
Physical function	66.67 ± 19.3	66.92 ± 17.0	66.30 ± 23.0	*p* = 0.906	70.0 ± 25.3	68.71 ± 14.4	*p* = 0.343
Social function	70.61 ± 20.6	69.44 ± 22.1	72.30 ± 18.5	*p* = 0.734	74.74 ± 17.7	70.63 ± 19.9	*p* = 0.450
FaCE	62.50 ± 24.8	57.07 ± 21.5	70.29 ± 27.4	*p* = 0.023*	74.17 ± 27.6	60.75 ± 20.3	*p* = 0.031*
SF36							
Physical function	78.91 ± 27.3	81.84 ± 23.6	74.62 ± 32.0	*p* = 0.537	79.72 ± 30.8	81.03 ± 22.9	*p* = 0.695
Role physical	67.58 ± 41.0	72.37 ± 39.3	60.58 ± 43.1	*p* = 0.247	69.44 ± 37.9	63.97 ± 43.2	*p* = 0.975
Bodily pain	73.02 ± 30.8	73.84 ± 28.9	71.81 ± 34.0	*p* = 0.965	80.11 ± 25.7	69.65 ±32.8	*p* = 0.289
General health	61.44 ± 21.3	62.08 ± 21.1	60.50 ± 21.9	*p* = 0.763	64.72 ± 15.9	62.00 ± 22.7	*p* = 0.862
Vitality	54.45 ± 18.5	54.21 ± 18.6	54.81 ± 18.7	*p* = 0.885	55.00 ± 19.1	53.53 ± 18.5	*p* = 0.780
Social function	76.95 ± 29.0	75.99 ± 26.2	78.37 ± 33.1	*p* = 0.306	81.25 ± 30.7	75.74 ± 26.8	*p* = 0.228
Role emotional	74.48 ± 38.8	71.05 ± 41.1	79.49 ± 35.4	*p* = 0.491	74.07 ± 38.9	73.53 ± 39.2	*p* = 0.911
Mental health	68.94 ± 19.4	67.47 ± 20.4	71.08 ± 18.0	*p* = 0.519	73.33 ± 18.0	67.41 ± 18.8	*p* = 0.243
PHQ-9	14.52 ± 3.8	14.39 ± 3.6	14.70 ± 4.1	*p* = 0.904	13.68 ± 3.0	14.62 ± 4.1	*p* = 0.456
ZUF-8	24.59 ± 6.2	24.09 ± 6.1	25.38 ± 6.29	*p* = 0.383	24.53 ± 6.7	24.41 ± 6.6	*p* = 0.971
Satisfied with local physical therapy							
Definitely not actually	3 (4.5%)	2 (5.1%)	1 (3.7%)	*X*^2^ = 1.84	1 (5.3%)	1 (2.9%)	*X*^2^ = 4.18
Not	9 (13.6%)	6 (15.4%)	3 (11.1%)	*p* = 0.765	2 (10.5%)	5 (14.3%)	*p* = 0.382
In general yes definitely	26 (39.4%)	14 (35.9%)	12 (44.4%)		6 (31.6%)	18 (51.4%)	
Yes	21 (31.8%)	14 (35.9%)	7 (25.9%)		6 (31.6%)	9 (25.7%)	
No statement	7 (10.6%)	3 (7.7%)	4 (14.8%)		4 (21.1%)	2 (5.7%)	
FP surgery							
No	53 (80.3%)	27 (69.2%)	26 (96.3%)	*X*^2^ = 7.39	19 (100%)	25 (71.4%)	*X*^2^ = 6.66
Yes	13 (19.7%)	12 (30.8%)	1 (3.7%)	*p* = 0.006*	0 (0%)	10 (28.6%)	*p* = 0.009*
Botulinum toxin							
No	51 (77.3%)	26 (66.7%)	25 (92.6%)	*X*^2^ = 13.27	16 (84.2%)	26 (74.3%)	*X*^2^ = 6.62
Yes	13 (19.7%)	13 (33.3%)	0 (0%)	*p* = 0.001*	1 (5.3%)	9 (25.7%)	*p* = 0.036*
Missing data	2 (3.0%)	0 (0%)	2 (7.4%)		2 (10.5%)	0 (0%)	
Conservative therapy							
Physical therapy	11 (16.7%)	4 (10.3%)	7 (25.9%)	*X*^2^ = 8.48	7 (36.8%)	2 (5.7%)	*X*^2^ = 18.50
Logopedics	17 (25.8%)	13 (33.3%)	4 (14.8%)	*p* = 0.132	2 (10.5%)	10 (28.6%)	*p* = 0.002*
Occupational therapy	1 (1.5%)	1 (2.6%)	0 (0%)		0 (0%)	0 (0%)	
No therapy	6 (9.1%)	2 (5.1%)	4 (14.8%)		4 (21.1%)	1 (2.9%)	
Several methods	30 (45.5%)	19 (48.7%)	11 (40.7%)		5 (26.3%)	22 (62.9%)	
Unknown	1 (1.5%)	0 (0%)	1 (3.7%)		1 (5.3%)	0 (0%)	

HB, House Brackmann score; FaCE, Facial Clinimetric Evaluation; FDI, facial disability index; FP, facial palsy; PHQ-9, Patient Health Questionnaire-9; SF-36, Short-Form Health Survey; VS, vestibular schwannoma; ^#^german Hauptschule, ^##^german; Realschule; *p-values indicate significance comparing pre- and postoperative patients by a Chi-square (*X*^2^) or Kruskal-Wallis test (H). Bold values are significant *p*-values.

The most frequent cause of FP was iatrogenic with 35/66 (53%, mainly after resection of vestibular schwannomas), followed by idiopathic FP (19/66, 28.1%). Less frequently infections (4/66, 6.1%) or a tumor itself were the cause of FP (5/66, 7.6%). However, the INTER and UNI groups differed significantly in the distribution of causes. While idiopathic causes were much more frequent in the UNI group, facial palsy of iatrogenic cause was leading in the INTER group (25/39, 64.1%), due to the also frequent resections of vestibular schwannomas performed in the local neurosurgical department. In *VS* patients, large *VS* (Koos 3 and 4: 36/40, 90%) clearly exceeded small tumors (Koos 2: 4/40, 10%). In addition, differences in the duration and recovery of FP emerged. Although patients in the INTER group had FP for longer on average, recovery was significantly worse than in the UNI group. Patients who were treated in the interdisciplinary consultation received significantly more often a surgical treatment regarding the facial palsy and more often botulinum toxin injections.

**Figure 2:**
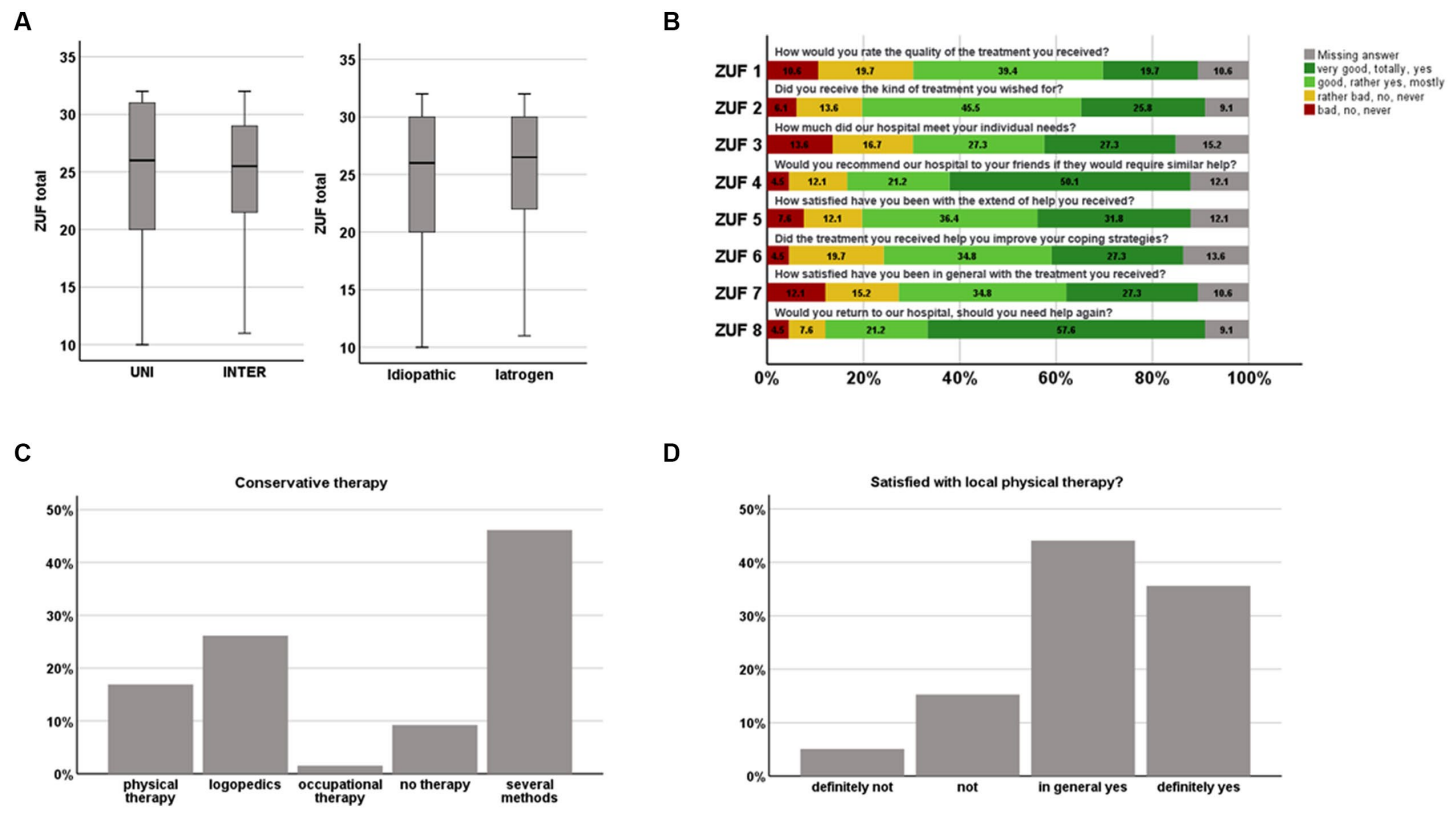
Patients satisfaction with facial palsy treatment. **(A,B)** Treatment in Tuebingen measured by the ZUF-8; and **(C,D)** of local physiotherapeutic/logopedic services.

Satisfaction with treatment did not differ between the groups (Figure 2). A total of 18.1% of patients were dissatisfied with the local physiotherapeutic/logopedic therapy. Similarly, 11/66 (16.7%; 7/39 INTER and 4/27 UNI) stated to be dissatisfied with the care at the university hospital. The ZUF-8 correlated significantly with the FDI-PF (*r* = 0.34, *p* = 0.011, Spearman’s), i.e., physical impairment due to FP. A correlation with patient age, degree of regression of the paresis, FaCE, FDI-SF or PHQ-9 could not be detected. However, an ANOVA revealed none of the above factors as predictive for the ZUF-8, including the FDI-PF. Also, satisfaction with local physiotherapeutic services did not correlate with the treatment satisfaction in Tuebingen measured by the ZUF-8 (*r* = 0.15, *p* = 0.292, Spearman’s). 3/11 patients (27.3%) were dissatisfied with the therapy in Tuebingen mentioned that they would have appreciated additional psychotherapy, 3/11 (27.3%) would have welcomed ophthalmologic care, and 7/11 (63.6%) gave other reasons (e.g., more information). Dissatisfied patients in the UNI group stated that they had only become aware of the facial outpatient service through this study and would have liked to have known about it earlier. Furthermore, some of the patients expressed the wish for better instructions for the training at home.

**Figure 3:**
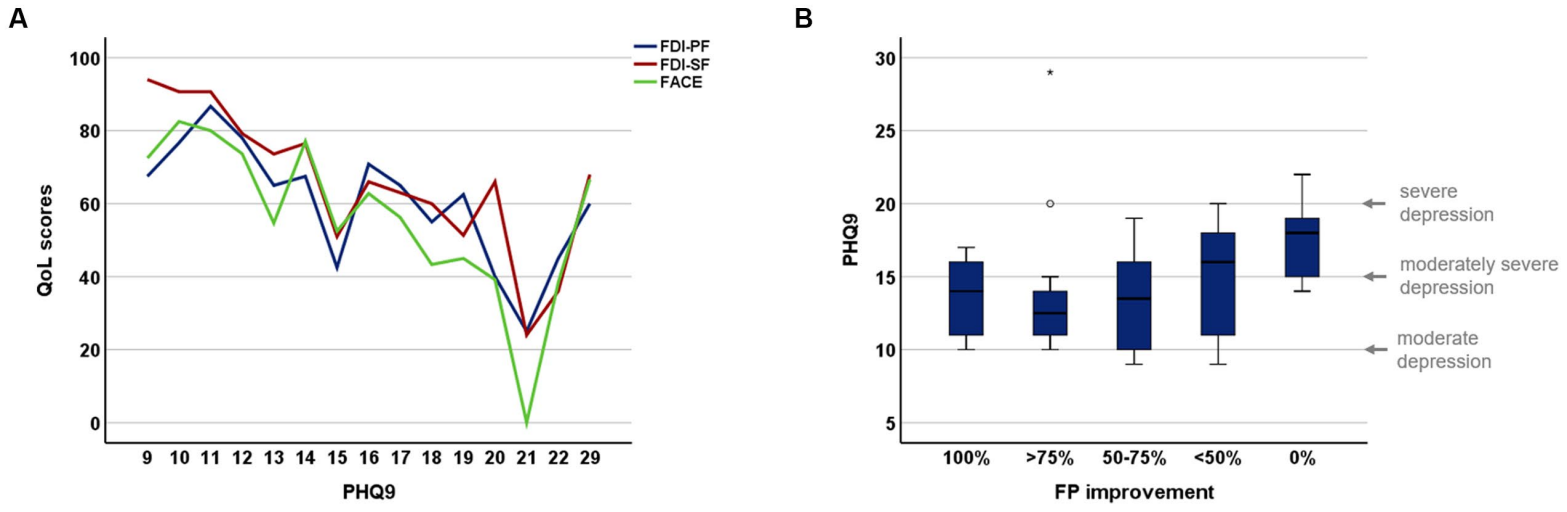
Depression scores in patients with facial palsy. **(A)** Correlation between PHQ-9 and facial specific QoL scores; **(B)** relation between PHQ-9 and facial palsy improvement.

There were no differences between the groups in QoL and level of depression as measured by the SF-36 and PHQ-9. However, an overall mild to moderate depression was observed in patients with FP (Figure 3). The degree of depression correlated with the FDI-PF (*r* = −0.45, *p* < 0.001), the FDI-SF (*r* = −0.70, p < 0.001), the FaCE (= − 0.50, *p* < 0.001) and the degree of regression of the paresis (*r* = 0.33, *p* = 0.008). In the group of *VS* patients, no correlation was found between QoL or depression scores and the size of VS.

### Idiopathic vs. iatrogen

Comparing patients with idiopathic and iatrogen FP, we found a significant difference for sex, distance to home, FP improvement, FaCE, and the frequency of FP-related surgeries and botulinum toxin injections. Notably, patients with idiopathic FP were more likely to come from the immediate vicinity of the university hospital, whereas patients with iatrogenic causes lived much more distantly. In contrast to the UNI/INTER comparison, there was no difference between the groups in the level of education or the duration of FP. However, analyses indicated a group difference in conservative therapies (e.g., logopedics) that was not found in the UNI/INTER comparison. Patients with iatrogenic FP were significantly more likely to use multiple therapies than patients with idiopathic FP.

### Reconstructive surgeries in facial palsy

A total of 13/66 (19.7%, 11 female) patients received surgical intervention due to FP, although one patient underwent an external procedure and did not provide any information about the procedure when surveyed. Therefore, this patient is not listed in Table 2 and will not be included in further analysis.

**Table 2:** Plastic and reconstructive surgeries of the study cohort.

	Procedure											
	CFNG (VII-VII)	HFGN (XII-VII)	MFGN (V-VII)	Lid chain	Static methods	Muscle transfer	Satisfaction	FDI-PF	FDI-SF	FACE	ZUF-8	PHQ9
1								65	52	50	28	15
2								75	84	56.7	11	12
3								70	48	41.7	24	19
4								55	32	16.7	21	19
5								70	76	68.3	25	16
6								75	84	68.3	30	13
7								60	92	80	24	9
8								50	32	31.67	24	19
9								65	20	-	-	18
10								95	96	85	31	17
11								45	56	23.3	19	16
12								55	60	43.3	31	18
Total	3	1	4	9	4	1		65.0 ± 13.5	61.0 ± 25.4	51.4 ± 22.6	24.2 ± 6.0	15.9 ± 3.2

CFNG, cross-facial nerve grafting; FDI-PF, Facial disability index physical function; FDI-SF, Facial disability index social function; HFGN, hypoglossal-facial nerve graft; MFGN, masseteric facial nerve transfer; dark green, very satisfied with procedure, green, satisfied with procedure, yellow, not satisfied with procedure.

The most common procedure among patients was implantation of a lid chain (9/12, 75%) (Table 2). Nerve reconstructive procedures included masseteric facial nerve transfer (MFNT; 4/12, 33.3%) most frequently, followed by cross-facial nerve grafting (CFNG; 3/12, 25%). Only one patient (8.3%)—who underwent external surgery—received reconstruction using a hypoglossal-facial nerve anastomosis (HFGN) in combination with CFNG. Overall, 11/12 (91.7%) patients were satisfied or very satisfied with the outcome of the procedure. Only one patient (8.3%) was dissatisfied after lid loading, as she developed a temporary hypospagma.

## Discussion

The occurrence of facial palsy is an impactful life event compromising patients’ mental and physical quality of life (4–6, 34). Treatment objectives should provide patients with the most holistic care possible, exhausting the full regenerative potential of the facial nerve. However, while many medical disciplines get in touch with FP patients, the disorder *“facial palsy”* usually represents only a minor topic in which only a few therapists specialize. This leads to an inadequate supply and possibly unspecific therapy (22). The present study examined treatment satisfaction and QoL in patients with FP in uni-and interdisciplinary treatment settings.

While overall satisfaction with FP treatment reached the ZUF-8 cut-off level of 24 points (29), no significant difference of satisfaction was found between UNI and INTER. This is contrary to the expectation that an interdisciplinary approach results in higher level of satisfaction (35). However, several factors may contribute to this discrepancy and suggest that interdisciplinary care could nevertheless preferable for the patient (20, 35, 36): (i) We found a significant difference between UNI and INTER regarding the cause of FP, duration and degree of recovery. While the UNI group exhibited a proportion of causes similar to that described in the literature (2), iatrogenic FP causes were overrepresented in the INTER group. This can be attributed to the direct affiliation of the interdisciplinary facial outpatient service with the Department of Neurosurgery in Tuebingen, which is a center for vestibular schwannomas. However, previous studies have shown that idiopathic FP generally recover better than palsies of iatrogenic origin and that improvement and chances for reinnervation may differ (14, 15), which could affect QoL and patient satisfaction. Vicente-Ruiz and Hontanilla (37) found that a longer duration of FP prior to reconstructive treatment was associated with decreased treatment satisfaction. Accordingly, it should further be evaluated whether different types of care are required for different causes of FP or whether interdisciplinary care should be offered in a standardized manner, e.g., after a certain period of time when the paresis has not improved (38, 39); (ii) While the overall response rate of the study was in good average compared to other questionnaire studies, there was a significantly better response rate in the INTER compared to the UNI group. This may be an indirect sign of patient satisfaction. However, a higher response rate due to the patient’s relationship with the researcher (KM), who also attends the interdisciplinary consultation, cannot be completely excluded (40); (iii) The distance to home was larger in the INTER group, suggesting that patients were willing to travel a greater distance for this treatment; (iv) Patients in the INTER group who were not completely satisfied with the treatment did not indicate a preference for an UNI setting, but wished the involvement of other disciplines (especially psychological and ophthalmological care). On the contrary, dissatisfied patients in the UNI group emphasized that they would have liked to have known about the option of specialized interdisciplinary consultation earlier.

Interdisciplinary treatment in this context offers the chance to treat a patient more holistically, as unidisciplinary treatment result in a narrower perspective on patient care. This has already been demonstrated in numerous oncological studies (41). With regard to the postoperative treatment of vestibular schwannomas, e.g., the control of the tumor as well as the treatment of FP can be performed simultaneously. Neurosurgeons are principally familiar with nerve surgery through the specialty of peripheral nerve surgery. However, the expertise in the area of the face is low. While HFGN are still performed, MFGN and CFNG are less common (20). Furthermore, there is often a lack of expertise in static procedures. Seeberger et al. (42) demonstrated in a nationwide study how often various types of procedures were preferred by different disciplines in FP. Interventions in the area of the mouth or eye were rarely performed by neurosurgeons. Through a joint consultation, the inhibition threshold in patients for plastic or maxillofacial surgery treatment can be lowered. While many patients may feel ashamed or embarrassed about seeking help from a plastic surgeon or there is a general societal stigma associated with cosmetic surgery, treatment by a “nerve surgeon” is more recognized; also by the health insurance companies, which are more often reluctant to cover costs of plastic surgery treatment although plastic surgery can be a valuable tool for improving the QoL of patients with FP, helping to restore their confidence and self-esteem (43, 44). In this context, our results show that significantly more patients in the INTER group received surgical treatment or injection with botulinum toxin compared with the UNI group. Almost all of these patients were satisfied with the surgical intervention. Similarly, Bradbury et al. found high levels of patient satisfaction after reconstructive surgery in FP (45).

Our results indicated that PHQ-9 scores were elevated in FP compared with the average normal population (46). In this context, previous studies showed that not only physical but also mental QoL was impaired. An U.S. study showed that patients with FP had depression in 47.7%, while only 5.1% of the control group had depression. Further investigations confirm considerable psychological distress in patients with facial palsy (47, 48). Though, there is disagreement whether the degree of FP determines the extent of QoL or depression (4, 5, 7, 48). While some studies have failed to demonstrate a correlation between these factors, other studies have shown a significant correlation, consistent with our findings. Regardless of this, psychological/psychiatric co-care of FP patients seems to be useful and was explicitly requested by patients. The effect of psychological counseling of FP patients to improve QoL and mental health has rarely been systematically investigated. However, a pilot study by Siemann et al. with a small number of cases suggests a positive effect of psychological counseling (49). Moreover, a significant reduction in anxiety and depression has been demonstrated as a result of psychological co-care in other medical conditions with a high psychosocial burden, such as oncological diseases (50, 51). Corresponding studies should also be performed for patients with FP in the future.

### Limitations

While the present study provides valuable insights into the outpatient treatment of FP patients, it is tempered by the single-center design which compromises generalizability of the results. A small cohort size reduces statistical power, precludes subgroup analyses and, therefore, reduces the informative value. The results may be influenced by the fact that only 66/108 patients participated in the survey and that the questionnaires were completed by the participants at different times after the therapy, so that there may be a recall bias. There is no information available on the extent to which the patients received psychological support (e.g., from relatives or friends) and whether antidepressant medication was taken. Finally, the absence of a matched UNI control group limits group comparability. In the future, these limitations should be considered in multi-center studies and should also take into account the composition of interdisciplinary teams.

## Conclusion

Treatment satisfaction of patients with FP depends on multifactorial aspects, whereby the present study could not demonstrate a difference in satisfaction between uni-and interdisciplinary consulting. However, in addition to treatment of physical impairments, psychological support should be provided. Surgical interventions should not be underestimated as potential treatment options in the absence of recovery, as they can improve quality of life and treatment satisfaction. Finally, the aspect of uni-and interdisciplinarity should be verified in future studies by addressing described limitations and using more homogeneous study groups.

## Data availability statement

The raw data supporting the conclusions of this article will be made available by the authors on reasonable request without undue reservation.

## Ethics statement

The studies involving humans were approved by ethical board of the University Hospital Tuebingen. The studies were conducted in accordance with the local legislation and institutional requirements. The participants provided their written informed consent to participate in this study.

## Author contributions

KM: Conceptualization, Formal analysis, Investigation, Methodology, Project administration, Supervision, Writing – original draft. LO: Data curation, Investigation, Methodology, Writing – review & editing. SW: Data curation, Writing – review & editing. EW: Data curation, Writing – review & editing. MG: Data curation, Writing – review & editing. HL: Methodology, Writing – review & editing. AD: Conceptualization, Resources, Writing – review & editing. MT: Conceptualization, Resources, Writing – review & editing. GN: Conceptualization, Resources, Writing – review & editing. RS: Conceptualization, Data curation, Investigation, Writing – original draft.

## References

[1] Hohman MH Hadlock TA. Etiology, diagnosis, and management of facial palsy: 2000 patients at a facial nerve center. Laryngoscope. 124:E283–93. doi: 10.1002/lary.24542 24431233

[2] Palsy Facial: Techniques for reanimation of the paralyzed face: Tzou, Chieh-Han John, Rodríguez-Lorenzo, Andrés: Amazon.de: Bücher. Available at: https://www.amazon.de/-/en/Chieh-Han-John-Tzou/dp/3030507831 (Accessed June 4, 2023).

[3] Hotton M Huggons E Hamlet C Shore D Johnson D Norris JH. The psychosocial impact of facial palsy: a systematic review. Br J Health Psychol. (2020) 25:695–727. doi: 10.1111/bjhp.12440 32538540

[4] Nellis JC Ishii M Byrne PJ Boahene KDO Dey JK Ishii LE. Association among facial paralysis, depression, and quality of life in facial plastic surgery patients. JAMA Facial Plast Surg. (2017) 19:190–6. doi: 10.1001/jamafacial.2016.1462, PMID: 27930763 PMC5469376

[5] Verhoeff R Bruins TE Ingels KJAO Werker PMN van Veen MM. A cross-sectional analysis of facial palsy-related quality of life in 125 patients: comparing linear, quadratic and cubic regression analyses. Clin Otolaryngol. (2022) 47:541–5. doi: 10.1111/coa.13934 35373461

[6] Machetanz K Lee L Wang SS Tatagiba M Naros G. Trading mental and physical health in vestibular schwannoma treatment decision. Front Oncol. (2023) 13:1152833. doi: 10.3389/fonc.2023.1152833, PMID: 37434979 PMC10332305

[7] Cross T Sheard CE Garrud P Nikolopoulos TP O’Donoghue GM. Impact of facial paralysis on patients with acoustic neuroma. Laryngoscope. (2000) 110:1539–42. doi: 10.1097/00005537-200009000-00024, PMID: 10983957

[8] Goldbrunner R Weller M Regis J Lund-Johansen M Stavrinou P Reuss D. Eano guideline on the diagnosis and treatment of vestibular schwannoma. Neurooncology. (2020) 22:31–45. doi: 10.1093/neuonc/noz153, PMID: 31504802 PMC6954440

[9] Kondziolka D Mousavi SH Kano H Flickinger JC Lunsford LD. The newly diagnosed vestibular schwannoma: radiosurgery, resection, or observation? Neurosurg Focus. (2012) 33:E8. doi: 10.3171/2012.6.FOCUS12192 22937859

[10] Wang SSY Machetanz K Ebner F Naros G Tatagiba M. Association of extent of resection on recurrence-free survival and functional outcome in vestibular schwannoma of the elderly. Front Oncol. (2023) 13:1153698. doi: 10.3389/fonc.2023.1153698, PMID: 37342182 PMC10277928

[11] Vakilian S Souhami L Melançon D Zeitouni A. Volumetric measurement of vestibular schwannoma tumour growth following partial resection: predictors for recurrence. J Neurol Surgery B Skull Base. (2013) 73:117–20. doi: 10.1055/s-0032-1301395, PMID: 23542125 PMC3424624

[12] Link MJ Lund-Johansen M Lohse CM Driscoll CLW Myrseth E Tveiten OV. Quality of life in patients with vestibular schwannomas following gross total or less than gross total microsurgical resection: should we be taking the entire tumor out? Clin Neurosurg. (2018) 82:541–7. doi: 10.1093/neuros/nyx245, PMID: 29554375

[13] Troude L Boucekine M Montava M Lavieille JP Régis JM Roche PH. Predictive factors of early postoperative and long-term facial nerve function after large vestibular schwannoma surgery. World Neurosurg. (2019) 127:e599–608. doi: 10.1016/j.wneu.2019.03.218, PMID: 30930324

[14] Urban E Volk GF Geißler K Thielker J Dittberner A Klingner C. Prognostic factors for the outcome of Bells’ Palsy: a cohort register-based study. Clin Otolaryngol. (2020) 45:754–61. doi: 10.1111/coa.13571, PMID: 32395899

[15] Hayler R Clark J Croxson G Coulson S Hussain G Ngo Q. Sydney facial nerve clinic: experience of a multidisciplinary team. ANZ J Surg. (2020) 90:856–60. doi: 10.1111/ans.15782, PMID: 32129559

[16] Rivas A Boahene KD Bravo HC Tan M Tamargo RJ Francis HW. A model for early prediction of facial nerve recovery after vestibular schwannoma surgery. Otol Neurotol. (2011) 32:826–33. doi: 10.1097/MAO.0b013e31821b0afd, PMID: 21527865

[17] Adegbite AB Khan MI Tan L. Predicting recovery of facial nerve function following injury from a basilar skull fracture. J Neurosurg. (1991) 75:759–62. doi: 10.3171/jns.1991.75.5.0759, PMID: 1919699

[18] Volk GF Klingner C Finkensieper M Witte OW Guntinas-Lichius O. Prognostication of recovery time after acute peripheral facial palsy: a prospective cohort study. BMJ Open. (2013) 3:e003007. doi: 10.1136/bmjopen-2013-003007 PMC3669721 23794548

[19] Silva MC Oliveira MT Azevedo-Santos IF DeSantana JM. Effect of proprioceptive neuromuscular facilitation in the treatment of dysfunctions in facial paralysis: a systematic literature review. Braz J Phys Ther. (2022) 26:100454. doi: 10.1016/j.bjpt.2022.100454, PMID: 36279766 PMC9597113

[20] Lassaletta L Morales-Puebla JM González-Otero T Moraleda S Roda JM Gavilán J. The experience of a facial nerve unit in the treatment of patients with facial paralysis following skull base surgery. Otol Neurotol. (2020) 41:E1340–9. doi: 10.1097/MAO.0000000000002902, PMID: 33492811

[21] Aronson S Applebaum SA Kelsey LJ Gosain AK. Evidence-based practices in facial reanimation surgery. Plast Reconstr Surg. (2023) 152:520e–33e. doi: 10.1097/PRS.0000000000010539, PMID: 37647378

[22] Szczepura A Holliday N Neville C Johnson K Khan Khan AJ Oxford SW. Raising the digital profile of facial palsy: national surveys of patients’ and clinicians’ experiences of changing UK treatment pathways and views on the future role of digital technology. J Med Internet Res. (2020) 22:e20406. doi: 10.2196/20406, PMID: 32763890 PMC7573702

[23] Györi E Przestrzelski C Pona I Hagmann M Rath T Radtke C. Quality of life and functional assessment of facial palsy patients: a questionnaire study. Int J Surg. (2018) 55:92–7. doi: 10.1016/j.ijsu.2018.04.061 29787803

[24] Kroenke K Spitzer RL Williams JBW. The PHQ-9: validity of a brief depression severity measure. J Gen Intern Med. (2001) 16:606–13. doi: 10.1046/j.1525-1497.2001.016009606.x, PMID: 11556941 PMC1495268

[25] Vanswearingen JM Brach JS. The facial disability index: reliability and validity of a disability assessment instrument for disorders of the facial neuromuscular system. Phys Ther. (1996) 76:1288–98. doi: 10.1093/ptj/76.12.1288 8959998

[26] Sun Y Kong Z Song Y Liu J Wang X. The validity and reliability of the PHQ-9 on screening of depression in neurology: a cross sectional study. BMC Psychiatry. (2022) 22:98. doi: 10.1186/s12888-021-03661-w, PMID: 35139810 PMC8827244

[27] Brazier JE Harper R Jones NMB O’Cathain A Thomas KJ Usherwood T. Validating the SF-36 health survey questionnaire: new outcome measure for primary care. Br Med J. (1992) 305:160–4. doi: 10.1136/bmj.305.6846.160 1285753 PMC1883187

[28] Schmidt J Lamprecht F Wittmann WW. Zufriedenheit Mit Der Stationaren Versorgung. Entwicklung Eines Fragebogens Und Erste Validitatsuntersuchungen. Psychother Psychosom Med Psychol. (1989) 39:248–55. 2762479

[29] Kriz D Nübling R Steffanowski A Wittmann WW Schmidt J. Patientenzufriedenheit in der stationären rehabilitation: psychometrische Reanalyse des ZUF-8 auf der Basis multizentrischer Stichproben verschiedener Indikation. Z Med Psychol. (2008) 17:67–79.

[30] Tarlov AR. The medical outcomes study: an application of methods for monitoring the results of medical care. JAMA J Am Med Assoc. (1989) 262:925–30. doi: 10.1001/jama.262.7.925 2754793

[31] Ware JE Sherbourne CD. The MOS 36-item short-form health survey (sf-36): I. Conceptual framework and item selection. Med Care. (1992) 30:473–83. doi: 10.1097/00005650-199206000-00002, PMID: 1593914

[32] Kahn JB Gliklich RE Boyev KP Stewart MG Metson RB McKenna MJ. Validation of a patient-graded instrument for facial nerve paralysis: the FaCE scale. Laryngoscope. (2001) 111:387–98. doi: 10.1097/00005537-200103000-00005, PMID: 11224766

[33] Volk GF Steigerwald F Vitek P Finkensieper M Kreysa H Guntinas-Lichius O. Facial disability index and facial clinimetric evaluation scale: validation of the German versions. Laryngorhinootologie. (2015) 94:163–8. doi: 10.1055/s-0034-1381999, PMID: 25089633

[34] Vargo M Ding P Sacco M Duggal R Genther DJ Ciolek PJ. The psychological and psychosocial effects of facial paralysis: a review. J Plast Reconstr Aesthetic Surg. (2023) 83:423–30. doi: 10.1016/j.bjps.2023.05.027, PMID: 37311285

[35] Butler DP Grobbelaar AO. Facial palsy: what can the multidisciplinary team do? J Multidiscip Healthc. (2017) 10:377–81. doi: 10.2147/JMDH.S125574, PMID: 29026314 PMC5626419

[36] Steinhäuser J Volk GF Thielker J Geitner M Kuttenreich AM Klingner CM. Multidisciplinary Care of Patients with facial Palsy: treatment of 1220 patients in a German facial nerve Center. J Clin Med. (2022) 11:427. doi: 10.3390/jcm11020427 35054119 PMC8778429

[37] Vicente-Ruiz M Hontanilla B. Measuring patient-reported outcomes after facial paralysis reconstruction surgery using the FACE-Q. Facial Plast Surg Aesthetic Med. (2023). doi: 10.1089/fpsam.2023.0022, PMID: 37406254

[38] Glass GE Tzafetta K. Optimising treatment of Bell’s Palsy in primary care: the need for early appropriate referral. Br J Gen Pract. (2014) 64:e807–9. doi: 10.3399/bjgp14X683041, PMID: 25452547 PMC4240155

[39] Butler DP Morales DR Johnson K Nduka C In: Tzou C-HJ Rodríguez-Lorenzo A, editors. Facial palsy. Cham: Royal College of General Practitioners (2019) 10.3399/bjgp19X706541 PMC6808583 31672833

[40] Holtom B Baruch Y Aguinis H Ballinger GA. Survey response rates: trends and a validity assessment framework. Hum Relat. (2022) 75:1560–84. doi: 10.1177/00187267211070769

[41] Pillay B Wootten AC Crowe H Corcoran N Tran B Bowden P. The impact of multidisciplinary team meetings on patient assessment, management and outcomes in oncology settings: a systematic review of the literature. Cancer Treat Rev. (2016) 42:56–72. doi: 10.1016/j.ctrv.2015.11.007, PMID: 26643552

[42] Seeberger S Schlattmann P Guntinas-Lichius O. Surgery for patients with facial palsy in Germany: a diagnosis-related-groups-based nationwide analysis, 2005–2019. Eur Arch Otorhinolaryngol. (2023) 281:451–9. doi: 10.1007/s00405-023-08259-4 37755497 PMC10764378

[43] Díaz-Aristizabal U Valdés-Vilches M Fernández-Ferreras TR Calero-Muñoz E Bienzobas-Allué E Aguilera-Ballester L. Effect of botulinum toxin type a in functionality, synkinesis and quality of life in peripheral facial palsy sequelae. Neurologia. (2021) 38:560–5. doi: 10.1016/j.nrl.2021.01.015 37437657

[44] Liu RH Yau J Derakhshan A Xiao R Hadlock TA Lee LN. Facial filler in facial paralysis: a prospective case study and multidimensional assessment. Facial Plast Surg Aesthetic Med. (2023). doi: 10.1089/fpsam.2023.0048, PMID: 37428541

[45] Bradbury ET Simons W Sanders R. Psychological and social factors in reconstructive surgery for hemi-facial palsy. J Plast Reconstr Aesthetic Surg. (2006) 59:272–8. doi: 10.1016/j.bjps.2005.09.003, PMID: 16673541

[46] Tomitaka S Kawasaki Y Ide K Akutagawa M Ono Y Furukawa TA. Stability of the distribution of patient health Questionnaire-9 scores against age in the general population: data from the National Health and nutrition examination survey. Front Psych. (2018) 9:390. doi: 10.3389/fpsyt.2018.00390, PMID: 30190687 PMC6115508

[47] Pouwels S Beurskens CHG Kleiss IJ Ingels KJAO. Assessing psychological distress in patients with facial paralysis using the hospital anxiety and depression scale. J Plast Reconstr Aesthetic Surg. (2016) 69:1066–71. doi: 10.1016/j.bjps.2016.01.021, PMID: 26952127

[48] Fu L Bundy C Sadiq SA. Psychological distress in people with disfigurement from facial palsy. Eye. (2011) 25:1322–6. doi: 10.1038/eye.2011.158, PMID: 21720412 PMC3194312

[49] Siemann I Sanches EE de Jongh FW Luijmes R Ingels KJAO Beurskens CHG. Psychological counselling in patients with a peripheral facial palsy: initial experience from an expert Centre. J Plast Reconstr Aesthetic Surg. (2022) 75:1639–43. doi: 10.1016/j.bjps.2021.11.079, PMID: 34975004

[50] Newell SA Sanson-Fisher RW Savolainen NJ. Systematic review of psychological therapies for cancer patients: overview and recommendations for future research. J Natl Cancer Inst. (2002) 94:558–84. doi: 10.1093/jnci/94.8.558 11959890

[51] Trijsburg RW Van Knippenberg FCE Rijpma SE. Effects of psychological treatment on cancer patients: a critical review. Psychosom Med. (1992) 54:489–517. doi: 10.1097/00006842-199207000-00011 1502290

